# Editorial: Chloroplast redox state: new insights into stress responses and acclimation

**DOI:** 10.3389/fpls.2023.1234946

**Published:** 2023-07-10

**Authors:** Mirko Zaffagnini, Maria Pilarska, Ewa Niewiadomska

**Affiliations:** ^1^ Department of Pharmacy and Biotechnology, University of Bologna, Bologna, Italy; ^2^ The Franciszek Górski Institute of Plant Physiology, Polish Academy of Sciences, Kraków, Poland

**Keywords:** chloroplast, redox state, thioredoxin (TRX) system, CBB cycle, stress responses

Redox reactions, a type of reaction involving a transfer of electron between two species, are fundamental for life on Earth. Universal carriers of reducing power such as pyridine nucleotides (NAD^+^/NADH, NADP^+^/NADPH) and thiol-based carriers (*e.g.*, thioredoxins, glutaredoxins, peroxiredoxins) are key regulators of multiple metabolic, signaling, and transcriptional processes in cells. This Research Topic brings together articles that deal with many aspects of redox regulation in plants, with special emphasis on reactions taking place in chloroplasts. It contains 4 papers, of which 3 are original research and one is a review.

Chloroplasts of higher plants are a site of the intensive turnover of NADP^+^/NADPH and thioredoxins (TRXs). In the light, reducing power is transferred from the photosynthetic electron transport system to TRXs, which then activate their target enzymes in the Calvin-Benson-Bassham (CBB) cycle. The TRX system has recently gained considerable attention as it is involved in maintaining the redox balance and regulating signal transduction.

In the first article, Zhou et al. characterize the typical TRX gene family in the wheat genome in regard to their distribution, duplication events, and phylogenic relationships. Several methodological approaches were employed to document the important role of *Triticum aestivum TRX* genes in the maintenance of redox homeostasis in control conditions and under stress. Among them were the functional annotation analyses of typical *TaTRX* genes by Gene Ontology and characterization of cis-elements in their promoters. On the example of the chosen TRXs gene (*TaTRX24*) response to osmotic stress was followed with WT and overexpressing line. Further on, publicly available RNA-seq samples from five tissues (root, stem, leaf, spike, and grain) were explored with a focus on the typical expression patterns of *TaTRX* genes in a broad spectrum of stress situations (drought, heat, salt, cold, infection with powdery mildew and stripe rust).

A study by García-Cañas et al. focuses the attention on two CBB enzymes, namely fructose-1,6-bisphosphatase and sedoheptulose-1,7-bisphosphatase (FBPase and SBPase, respectively), which are validated targets of light-dependent regulation mediated by TRXs. To assess the relative importance of their activity in ensuring photoautotrophism, the authors followed an interesting approach by complementing the cyanobacterial SBPase/FBPase mutant with orthologs from higher plants. The expression of both plant enzymes restored photoautotrophic growth, but at reduced rates and only under low light conditions. Improved growth of the complemented strain was achieved when plant f-type TRX was co-expressed with CBB enzymes, indicating that an efficient modulation of the redox state of plant enzymes is crucial to allow proper functioning under light conditions. Overall, this study demonstrates that plant proteins can be reduced by the cyanobacterial TRX system, and opens the possibility to use this complementation approach as a reliable tool to analyze *in vivo* the specificity of the TRX system toward redox-regulated plant proteins.

The review by Meloni et al. provides a comprehensive survey of three CBB cycle enzymes involved in the regeneration of the substrate of the ribulose-1-5-bisphosphate carboxylase/oxygenase (Rubisco). These enzymes are ribose-5-phosphate isomerase (RPI), ribulose-5-phosphate epimerase (RPE), and phosphoribulokinase (PRK). The authors retrieve from the literature the current knowledge on the structural and catalytic properties of these photosynthetic enzymes, highlighting the molecular basis of enzyme functioning in the context of the chloroplast stroma. In addition, the authors provide information on regulatory mechanisms involving metabolites or post-translational modifications such as oxido-reduction of protein cysteines and phosphorylation at serine and threonine residues. These redox and phospho-dependent regulations are of particular interest since the three enzymes have been identified as potential targets by proteomic-based approaches. Modifications of specific residues can thus alter the function of the protein in response to both physiological and stress conditions, with consequences for metabolic flux and thus for Rubisco substrate regeneration. As future perspectives, the authors discuss possible strategies to be applied as future research lines aimed at improving photosynthetic efficiency and plant productivity.

The reducing power carriers are also necessary for the action of non-photosynthetic plastids. Noteworthy, plastids in non-photosynthetic tissue are also capable of producing NADPH from glucose via the oxidative pentose phosphate pathway (OPPP). This reducing power is necessary for nitrite reduction, antioxidant function and several other pathways.

The paper by Née et al. demonstrates that the entry point of OPPP in non-photosynthetic plastids, namely glucose-6-phosphate dehydrogenase (G6PDH) reaction, primarily involves G6PDH2 and G6PDH3 isoforms in the model plant *Arabidopsis thaliana*. *In vitro* analysis revealed the activity of both enzymes undergoes a dithiol/disulfide regulatory interchange with m-type TRX being found to be the most efficient. This regulation is crucial for root growth, as shown with the *trxm1trxm2trxm4* triple mutant. Such redox activation is important, for example, for stimulation of G6PDH activity under salinity, in which the G6PDH2 contributes predominantly.

In conclusion, we believe that this Research Topic indeed provides new information about a number of proteins involved in redox regulatory networks in the chloroplast, either thioredoxins or carbon-related metabolic enzymes. It is our hope that in the near future, it will be possible to cover additional topics, in particular, in the field of light-dependent reactions of photosynthesis, as components of the photosynthetic apparatus along with regulatory proteins are likely regulated through redox mechanisms.

## Author contributions

All authors listed have made a substantial, direct, and intellectual contribution to the work and approved it for publication.

